# Medicinal Plants in Management of Type 2 Diabetes and Neurodegenerative Disorders

**DOI:** 10.1155/2015/686872

**Published:** 2015-03-19

**Authors:** Gan Siew Hua, Mohammad Amjad Kamal, Marcelo M. S. Lima, Mohamed Ibrahim Khalil, Visweswara Rao Pasupuleti, Gjumrakch Aliev

**Affiliations:** ^1^Human Genome Centre, School of Medical Sciences, Universiti Sains Malaysia, 16150 Kubang Kerian, Kelantan, Malaysia; ^2^King Fahd Medical Research Center, King Abdulaziz University, P.O. Box 80216, Jeddah 21589, Saudi Arabia; ^3^Laboratorio de Neurofisiologia, Departamento de Fisiologia, Universidade Federal do Parana (UFPR), 81531-980 Curitiba, Brazil; ^4^Department of Biochemistry and Molecular Biology, Jahangirnagar University, Savar, Dhaka 1342, Bangladesh; ^5^Faculty of Agro Based Industry, Universiti Malaysia Kelantan, Jeli Campus, Locked Bag No. 100, 17600 Jeli, Kelantan, Malaysia; ^6^School of Health Science and Healthcare Administration, University of Atlanta, E. Johns Crossing, Suite 175, Johns Creek, GA 30097, USA

With advancement in modern medical sciences, a variety of major diseases such as type 2 diabetes and neurodegenerative disorders (ND) including Alzheimer's disease, Parkinson's disease, and the mood disorders still prevail. Medicinal plants have the potential to prevent, control, or cure some of these diseases. For example, some plants have constituents useful as antiaging potentials due to the presence of antioxidants. Many of the plants have high medicinal values to be further explored. However, determining specific components having specific pharmacological activity and their clinical effects still remains a challenge. This issue is a compilation of 10 intriguing papers focusing on medicinal plants with potential for management of diabetes and ND.

Berberine (Ber) is an isoquinoline derivative alkaloid isolated from several Chinese medicines such as* berberidis radix*,* phellodendri chinensis cortex*,* coptidis rhizoma,* and* mahoniae caulis*. In recent decades, much attention has been placed on its significant antidiabetic activities. Z. Wang et al. conducted a research to improve the problem posed by the plant's low solubility and poor membrane permeability by incorporating nanosuspension technology. Due to the enhanced absorption posed by the technology, they concluded that Ber has good potential as a novel potential antidiabetic agent for both functional food and pharmaceutical purposes.

The paper by N. Giribabu et al. focused on* Vitis vinifera *(Linn.) which belongs to Vitaceae family. Besides having antidiabetic activity,* V. vinifera* also has a wide range of pharmacological activities such as inhibition of platelet aggregation and low density lipoprotein oxidation, antioxidant, antimicrobial, and anticancer activities. In their research paper, the authors explored the potential of the seed extract to protect against diabetes-induced liver damage for the first time. The authors found that it has the ability to confer near normal activity levels for various key enzymes involved in liver carbohydrate metabolism in diabetes. In addition, liver histology findings following administration of* V. vinifera* seed ethanolic extract were also very promising. The authors hypothesized that these positive changes may be due to decrease in liver oxidative stress in diabetes and suggested that the seed extract of a Muscat variety of* V. vinifera* helps in liver protection in diabetes.

N. Giribabu et al. investigated* Centella asiatica*, an herb traditionally used to improve memory on prevention of hippocampus dysfunction in diabetic rats. They reported that administration of* C. asiatica *leaf aqueous extract to diabetic rats maintained near normal ATPases activity levels and prevented the increase in the levels of inflammatory and oxidative stress markers in the hippocampus. The authors suggested that* C. asiatica *leaf protects the hippocampus against diabetes-induced dysfunction which could help to preserve memory in diabetes.

*P. niruri *is another medicinal plant reported to possess antidiabetic and kidney protective effects. N. Giribabu et al. investigated the phytochemical constituents and* in vitro *antioxidant activity of* P. niruri *leaf aqueous extract on oxidative stress and antioxidant enzymes levels in diabetic rat kidney. They found that administration of* P. niruri *leaf aqueous extract for 28 consecutive days prevented the increase in the amount of lipid peroxidation product, malondialdehyde, and the diminution of superoxide dismutase, catalase, and glutathione peroxidase activity levels in the kidney of diabetic rats. In addition,* P. niruri *leaf aqueous extract also exhibited* in vitro *antioxidant activity while phytochemical screening of the extract indicated the presence of polyphenols. The authors concluded that* P. niruri *leaf extract protects the kidney from oxidative stress induced by diabetes.

In another paper, I. Aziz et al. explored the potential of Spirulina platensis (a commonly used nutritional supplement due to its high protein and antioxidant content) as a neuroprotective agent in both experimental focal and global cerebral ischemia-reperfusion injuries in rats. They found that pretreatment with Spirulina significantly improved the rats' locomotor function and ameliorated the histological changes and neurological deficits thus suggesting that Spirulina possesses potential benefits in improving hind limb locomotor function and reducing morphological damage to the spinal cord which could offer alternatives to the limited management available for spinal cord injury.

S. N. Talukdar et al. reviewed the potential of* Momordica dioica* (a perennial, dioecious, cucurbitaceous climbing creeper), native to Asia for its phytochemical, ethnobotanical, phytotherapeutical, and pharmacological properties based on traditional view (including Ayurveda) along with recent scientific observations. Their effort can help many researchers justify the dynamic ethnobotanical and phytotherapeutical roles of several plants for future research as well as stimulating new ideas for therapeutic roles of* Momordica dioica *against diabetes, cancer, ND, and other life threatening disorders.

The* in vitro* and* in vivo *effects of* Cuscutae Semen* (CS) (a widely used traditional herbal medicine) on neurotoxicity were investigated by M. Ye et al. The* in vitro* assay indicated that CS attenuated 1-4-methyl-4-phenylpyridinium- (MPTP-) induced cell death, increased reactive oxygen species generation, and activated glutathione peroxidase.* In vivo*, immunohistochemistry assay for tyrosine hydroxylase revealed a significant loss of nigral dopamine neurons with parallel activation of the microglia and increased production of reactive oxygen species was also observed in the substantia nigra and striatum. The MPTP-induced loss of nigral dopamine neurons was, however, partly inhibited by CS suggesting that CS may be useful for the treatment of ND such as Parkinson's disease which warrants further investigation.

Another medicinal plant,* Codonopsis lanceolata *(*C. lanceolata*), has previously been employed for lung inflammatory diseases such as asthma, tonsillitis, and pharyngitis. J. B. Weon et al. evaluated the effects of fermented* C. lanceolata* on learning and memory impairment induced by scopolamine by using the Morris water maze and passive avoidance tests. To elucidate the possible mechanism of cognitive enhancing activity, the authors also measured acetylcholinesterase activity, brain-derived neurotrophic factor, and cyclic AMP response element-binding protein expression in the brain of mice. They found that the administration of fermented* C. lanceolata* reduced scopolamine-induced memory impairment in the Morris water maze and passive avoidance tests. Interestingly, it also inhibited acetylcholinesterase activity while the level of cyclic AMP response element-binding protein phosphorylation and neurotrophic factor expression in hippocampal tissue were significantly increased indicating that fermented* C. lanceolata *can ameliorate scopolamine-induced memory deficits in mouse and may be an alternative agent for the treatment of Alzheimer's disease in the future.

M. Rasool et al. reviewed the use of various phytochemicals in the treatment of ND. Many of the phytochemicals could be used as promising therapeutic agents because many have anti-inflammatory, antioxidative, and anticholinesterase activity. The review covers the various researches related to phytochemicals in management of ND.

Honey is the only insect-derived natural product with therapeutic, traditional, spiritual, nutritional, cosmetic, and industrial value. In addition to having excellent nutritional value, honey is a good source of physiologically active natural compounds, such as polyphenols. Unfortunately, there are very few current researches investigating the nootropic and neuropharmacological effects of honey, and these are still in their early stages. M. M. Rahman et al. reviewed the nootropic effects of honey, such as memory-enhancing effects, as well as neuropharmacological activities including anxiolytic, antinociceptive, anticonvulsant, and antidepressant activities. The review may help impact the use of honey for specific ND, apoptosis, necrosis, neuroinflammation, synaptic plasticity, and behavior-modulating neural circuitry in the future.

Overall, by compiling these papers, we hope to enrich both readers and researchers alike on the vast potential of natural products for the treatment of diabetes and ND for which treatment based on modern medicine is still lacking.

*Gan Siew Hua* *Gan Siew Hua* *Mohammad Amjad Kamal* *Mohammad Amjad Kamal* *Marcelo M. S. Lima* *Marcelo M. S. Lima* *Mohamed Ibrahim Khalil* *Mohamed Ibrahim Khalil* *Visweswara Rao Pasupuleti* *Visweswara Rao Pasupuleti* *Gjumrakch Aliev* *Gjumrakch Aliev*

